# *Brevibacillus laterosporus*, a Pathogen of Invertebrates and a Broad-Spectrum Antimicrobial Species 

**DOI:** 10.3390/insects4030476

**Published:** 2013-09-05

**Authors:** Luca Ruiu

**Affiliations:** Dipartimento di Agraria, University of Sassari, Via E. De Nicola, 07100 Sassari, Italy; E-Mail: lucaruiu@uniss.it; Tel.: +39-079-229326; Fax: +39-079-229329

**Keywords:** *Brevibacillus laterosporus*, entomopathogenic bacteria, insecticide, nematicide, biopesticide, pests, biological control, antimicrobial, bioremediation

## Abstract

*Brevibacillus laterosporus*, a bacterium characterized by the production of a unique canoe-shaped lamellar body attached to one side of the spore, is a natural inhabitant of water, soil and insects. Its biopesticidal potential has been reported against insects in different orders including Coleoptera, Lepidoptera, Diptera and against nematodes and mollusks. In addition to its pathogenicity against invertebrates, different *B. laterosporus* strains show a broad-spectrum antimicrobial activity including activity against phytopathogenic bacteria and fungi. A wide variety of molecules, including proteins and antibiotics, have been associated with the observed pathogenicity and mode of action. Before being considered as a biological control agent against plant pathogens, the antifungal and antibacterial properties of certain *B. laterosporus* strains have found medical interest, associated with the production of antibiotics with therapeutic effects. The recent whole genome sequencing of this species revealed its potential to produce polyketides, nonribosomal peptides, and toxins. Another field of growing interest is the use of this bacterium for bioremediation of contaminated sites by exploiting its biodegradation properties. The aim of the present review is to gather and discuss all recent findings on this emerging entomopathogen, giving a wider picture of its complex and broad-spectrum biocontrol activity.

## 1. Introduction

**Figure 1:**
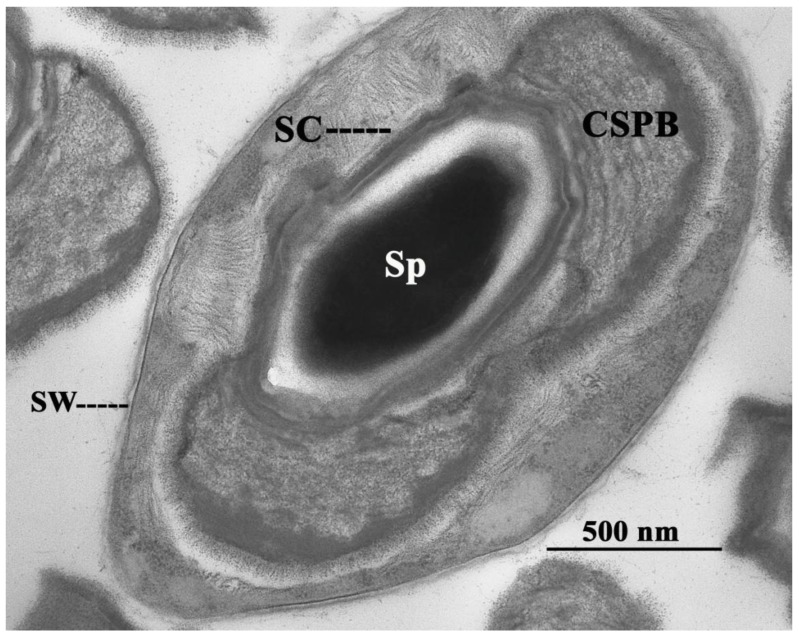
*Brevibacillus laterosporus* sporangium. SW, sporangium wall; Sp, spore; SC, spore coat; CSPB, canoe-shaped parasporal body.

*Brevibacillus laterosporus* Laubach is a rod-shaped, endospore-forming bacterium morphologically characterized by the production of a typical canoe-shaped parasporal body (CSPB) firmly attached to one side of the spore, which determines its lateral position in the sporangium (Figure 1). 

It is an ubiquitous species that has been isolated from a wide range of materials including soil [1], gemstones [2], lahar [3], fresh water [4], sea water [5], insect bodies [6], leaf surfaces [7], locust beans [8]; compost [9], milk [10], cheese [11], honey [12], starchy foods [13], gelatin-factory effluents [14], animal hide and wool [15], quails [16].

The significance of its relationships with insects was noted after it was first isolated at the beginning of the 20th century by White (1912) who was conducting studies on the bacterial community of honeybees affected by European foulbrood. Among secondary invaders in the disease, he isolated a new species that he named *Bacillus orpheus* White [6]. A detailed description of the bacterium was given in 1917 by McCray, who isolated different spore-forming bacteria of an apiary [17]. However, a year earlier, *Bacillus laterosporus* had been named and described by Laubach, who isolated a different strain from fresh water [4]. Later on, given the correspondence between the two isolates, the second name had priority and was chosen to designate this bacterium [18]. In 1946 Steinhaus listed it among spore-forming entomogenous species [19]. As a result of recent taxonomic studies based on 16S rRNA sequence analysis, this species has been relocated in the new genus *Brevibacillus* within the *Brevibacillus brevis* cluster [20]. 

The biocontrol potential of this entomopathogenic species has been reported against insects in different orders including Coleoptera, Lepidoptera, Diptera and against nematodes and mollusks [21]. Non-pathogenic effects towards non-target species have also been noted for entomopathogenic strains.

*B. laterosporus* is also an occasional inhabitant of honeybee bodies. It has been isolated from the digestive tract of healthy foraging adult workers, although its occurrence was sporadic in the examined samples [22]. Occasionally, it has been isolated from diseased honeybee larvae, especially those affected by European foulbrood (EFB). However, it has been considered to be a probiotic or just a secondary bacterial invader [23,24]. It is listed among bacteria that do not cause European foulbrood, but can be helpful in its diagnosis because it influences the odor and consistency of the dead brood, together with *Paenibacillus alvei* Cheshire and Cheyne, *Enterococcus faecalis* (Andrewes and Horder) Schleifer and Kilpper-Balz, *Bacterium eurydice* White and *Paenibacillus apiarius* (Katznelson) Nakamura [25].

In addition to its pathogenicity against invertebrates, different strains of *B. laterosporus* show a broad-spectrum antimicrobial activity especially against bacteria and fungi. A wide variety of molecules, including proteins and antibiotics, have been associated with the observed pathogenicity and mode of action. A recent whole genome sequencing of this species revealed its potential to produce polyketides, nonribosomal peptides, and toxins [26,27]. This species is also listed among probiotics for mammals and birds [28,29], and specific antibiotics derived from it have found use in medicine [30].

The aim of the present review is to gather and discuss all recent findings on this entomopathogenic, nematicidal, molluscicidal and antimicrobial species, giving a wider picture of its complex and broad‑spectrum biocontrol actions.

## 2. The Canoe-Shaped Parasporal Body (CSPB) and Other Parasporal Bodies (PBs)

The main morphological feature characterizing *B. laterosporus* is an aspect that has over time attracted the interest of different researchers. In the middle of the 20th century, Hannay conducted observations on fixed and embedded sporulating cells and spores under light and electron microscopy, coming to the conclusion that the “canoe-shaped parasporal body is formed before the completion of sporulation and persists still attached to the spore after sporulation” because it is “part of the spore” [31]. 

Different hypotheses have been made on the possible roles and functions of these bodies that could act as storage units if spores germinate in deficient media, might assist spore germination or might be an accessory wall or just a deformation of the spore wall. However, a specific function has not been confirmed so far. Fitz James and Young (1958) analyzed the chemical composition of the canoe-spore-coat structure, after developing a method for its separation from the spore protoplast [32]. According to their study, the main components of the CSPB are phosphorous and nitrogen. In addition to abundant phosphates, proteinaceous material was recovered through alkaline extraction in the presence of reducing agents (alkali-thioglicollate) disrupting S-S bonds. The results of combined morphological and chemical analyses suggest that the phosphorus component is part of a lamellar structure while extractable proteins would correspond to the matrix of the “canoe”.

Other parasporal bodies (PBs), distinct from the CSPB, were noted inside the sporangium by Montaldi and Roth (1990) who identified a second type, globular to angular in shape, and a third type, striated with alternating parallel bands [33]. The possible function of these structures was not provided, but later on the role of *B. laterosporus* parasporal bodies with variable size and shapes in the entomocidal activity against Diptera emerged.

## 3. Pathogenicity for Invertebrates

The pathogenicity of various *Brevibacillus laterosporus* strains has been confirmed for different invertebrates like insects, nematodes and mollusks [21]. Depending on the target, the toxic effects have been observed after contact or ingestion of diverse fractions produced at different stages in the bacterial growth cycle, from the vegetative to the sporulation phase. Specific protein toxins have been identified and their mode of action has been explored. However, many aspects still need to be clarified and further investigation is needed. 

### 3.1. Insects

#### 3.1.1. Coleoptera

Different strains of *B. laterosporus* were employed in experiments with various species of Coleoptera. Initially, certain strains were found to be toxic to larvae of the coleopteran species *Leptinotarsa decemlineata* (Say) (Coleoptera: Chrysomelidae) and *Lasioderma serricorne* (Fabricius) (Coleoptera: Anobiidae) [34,35].

Similarly, some isolates were found to be toxic to the red palm weevil *Rhynchophorus ferrugineus* (Olivier) (Coleoptera: Curculionidae) [36] and the boll weevil *Anthonomus grandis* Boheman (Coleoptera: Curculionidae) [1]. Slight toxicity was observed under laboratory conditions by topical applications of a bacterial mixture including *B. laterosporus* against adults of the snout-beetle *Gonipterus scutellatus* Gyllenhal (Coleoptera: Curculiionidae), a defoliator of *Eucalyptus* plantations [37].

A *B. laterosporus* isolate was also recorded to be highly toxic in comparative bioassays with different *Bacillus thuringiensis* Berliner isolates against *Tenebrio molitor* L. (Coleoptera: Tenebrionidae) larvae. An LC_50_ value of 3.388 × 10^6^ spores/mL was determined [38]. 

The molecular characterization of certain coleopteran-active strains demonstrated the production of insecticidal protein toxins showing similarity to those produced by *B. thuringiensis*. These toxins are secreted and released in the culture broth and consequently toxicity is associated with the culture supernatant, which is particularly active against Diabrotricine beetles also known as corn rootworms (*Diabrotrica* spp.). These “insecticidal secreted proteins” (ISPs) are also toxic to other coleopteran species such as *Leptinotarsa* spp. and *Anthonomus* spp., but they do not show any toxicity against Lepidoptera. The protein nature of these toxins was at first determined by the observation that supernatant heat treatments caused a loss of toxicity, whereas retention of activity was observed after ammonium sulfate precipitation. Novel ISPs were designated as ISP1A and ISP2A and the DNA sequences encoding them was determined [39]. Interestingly, a primary ISP protein is toxic only if ingested by the insect in combination with its complementary ISP protein, and no significant toxicity is observed when an ISP protein is used in isolation. ISP1A and ISP2A have molecular weights of 95–100 kDa and 45–50 kDa, respectively, as determined by standard 8%–10% SDS-PAGE, and show high homology with *B. thuringiensis* vegetative insecticidal proteins (VIP), so that they are now listed as Vip1Da1 and Vip2Ad1, respectively [40,41]. Other vegetative insecticidal proteins with a molecular weight of about 80 kDa and 40 kDa have been discovered and named MIS (=Vip1Ba1) and WAR (=Vip2Ba1), respectively [42]. Their genes were cloned and expressed in *B. thuringiensis* as well as inserted into plants using *Agrobacterium tumefaciens* (Smith and Townsend) Conn as transformation agent. These toxins are also highly toxic to larvae of *Diabrotica* spp..

#### 3.1.2. Lepidoptera

The results of *B. laterosporus* bioassays against Lepidoptera are controversial as a consequence of a considerable variability among different isolates. *B. laterosporus* strains show variable toxicity levels to the velvetbean caterpillar *Anticarsia gemmatalis* Hubner (Lepidoptera: Noctuidae), while the same strains were not toxic to the fall armyworm *Spodoptera frugiperda* (J.E. Smith) (Lepidoptera: Noctuidae) [1]. Moreover, some strains toxic against mosquitoes failed to show significant pathogenicity against larvae of the tobacco budworm *Heliothis virescens* F. (Lepidoptera: Noctuidae), the cabbage looper *Trichoplusia ni* Hubner (Lepidoptera: Noctuidae), the european corn borer *Ostrinia nubilalis* Hubner (Lepidoptera: Pyralidae), and the tobacco hornworm *Manduca sexta* (Linnaeus) (Lepidoptera: Sphingidae) [34,43].

*B. laterosporus* was isolated from larvae of the codling moth *Cydia pomonella* L. (Lepidoptera: Tortricidae), collected from leaves of apple trees in Turkey [44]. Remarkably, among other bacterial species isolated from the same sites, it exhibited the most toxicity. 

#### 3.1.3. Mosquitoes and Black Flies

The earliest reports on the insecticidal potential of *B. laterosporus* involved studies with mosquitoes. The pathogenicity of 29 strains against *Culex quinquefasciatus* Say (Diptera: Culicidae) and *Aedes aegypti* (L.) (Diptera: Culicidae) larvae and for the black fly *Simulium vittatum* Zetterstedt (Diptera: Simuliidae) was demonstrated by Favret and Yousten [43], although the level of toxicity recorded was about 1,000 times less than the mosquitocidal strain of *B. thuringiensis* serovar *israelensis* H14. Unlike the previously described cases of Coleoptera, the pathogenicity for mosquitoes was not associated to the culture supernatant, but to the cell mass and the effects were maintained after bacteria were killed by UV irradiation, which supported the hypothesis of a toxin mediated process. Toxicity was associated with stationary-phase cells or to sporulated cultures, as confirmed by subsequent studies [34]. In line with this, a recent study with *B. laterosporus* indicated that spores with their CSPB structures were the main location of toxins active against *A. aegypti* [45].

Following the discovery of *B. laterosporus* Strains 615 and 921 from Russia, the production of cytoplasmic crystalline inclusions of various shapes and sizes [46] and their roles in insecticidal activity against mosquitoes were reported [47]. In this case, the insecticidal action was also immediately associated with sporulated cells rather than the culture supernatant. An overproduction of crystalline inclusions was obtained by growing *B. laterosporus* on Nickerson agar instead of Nickerson broth. Crystalline inclusions were purified from spores, their protein concentration determined and their insecticidal activity confirmed against *A. aegypti* and *Anopheles stephensi* Liston (Diptera: Culicidae). The observed median lethal concentration (LC_50_) was 3.0 ng mL^−1^ and 5 ng mL^−1^, respectively. These levels of toxicity are comparable to those known for the highly toxic mosquitocidal strains of *B. thuringiensis* subsp. *israelensis* [48]. Major proteins with a molecular weight of 68 kDa and 130 kDa were identified in the crystal fractions from these *B. laterosporus* strains [49]. In a recent study, a mosquitocidal activity was found in crystal-forming strains of *B. laterosporus* after incapsulation of spores and crystal mixtures in the protozoa *Tetrahymena pyriformis* GL and *Entamoeba moshkovskii* Chalaia. Interestingly, the spore-crystal preparations were not toxic to protozoa cells [50].

#### 3.1.4. House Fly

First evidence of the susceptibility of the house fly *Musca domestica* L. (Diptera: Muscidae) to *B. laterosporus* was reported in 2006 by Ruiu *et al.* [51], who employed sporulated cultures of the bacterium. The observed signs and symptoms of *B. laterosporus* infection on the house fly are similar to those caused by other entomopathogenic bacteria, such as *B. thuringiensis*, on various insect targets [52]. In addition to lethal effects on adults and larvae exposed to a treated diet, the effects of sub-lethal concentrations were studied. These included a significant increase in larval development time and reductions in pupal weight, emergence rate, adult fecundity and longevity. More detailed studies on the effects of different bacterial fractions on house flies, corroborated the results of Favret and Yousten [43], who reported that vegetative cells become toxic to mosquitoes in the stationary phase, and that toxicity increases and is maintained after sporulation [45]. In the case of house flies, spores of the toxic strain were identified as the main source of toxicity. A nearly pure fraction of spore‑coats attached to the CSPBs, obtained through protoplast disruption with glass beads, showed toxicity against flies, and a protein fraction they contain was directly associated to the insecticidal effects. Carrying toxins attached firmly to the viable spore can be seen as an advanced strategy of a bacterium like *B. laterosporus* in comparison to other bacteria like *B. thuringiensis* whose full insecticidal action relies on the adventitious co-ingestion of spores and free cytoplasmic toxin crystals. Observations on insect behavior and midgut ultrastructure after ingestion of *B. laterosporus* spores have been conducted to study the subsequent pathological and histopathological events [53]. For this purpose, larvae were dissected at different time intervals after treatment and the mid-midgut portion dissected and fixed for tissue analysis under transmission electron microscopy (TEM). In comparison to healthy insects, treated ones rapidly reduced their feeding and growth rate, becoming sluggish before paralysis and death. At the same time, the midgut epithelium deteriorated and showed progressively more dramatic alterations including endoplasmic reticulum deformation, mitochondria alteration, microvilli disruption, cytoplasm vacuolization and general disorganization with cell lysis. Degenerative changes also affected the midgut muscular sheath and connective tissue, thus suggesting a correlation with a behavioral symptomatology, ending with insect paralysis. Remarkably, affected and unaffected midgut epithelial cells were often adjacent to one another, which suggests a cell‑specific susceptibility related to the interaction of bacterial toxins with the epithelial cell membranes. Similar cellular alterations were observed on fly adults [54].

Experimental formulations based on *B. laterosporus* spores have been developed and employed in application trials on house fly breeding sites in dairy farms, where a significant suppression of adult emergence from manure was obtained [55,56]. 

Another worthy aspect that has been investigated in the evaluation of *B. laterosporus* as a biological control agent against flies, is its safety towards non-target species, with special regard to the hymenopteran house fly pupal parasitod *Muscidifurax raptor* Girault and Sanders (Hymenoptera: Pteromalidae) [57] and to the honey bee *Apis mellifera* L. (Hymenoptera: Apidae) [58].

### 3.2. Nematodes

*B. laterosporus* was found to be a biocontrol agent of parasitic nematodes after a nematicidal compound from spores of certain strains was discovered to inhibit egg hatching and larval development of roundworms. The toxin, isolated by HPLC, was a heat stable, low molecular weight protein of approximately 2,900 daltons, characterized by UV absorbance at 205, 220 and 268 nm [59]. Nematicidal activity was reported for parasitic nematodes of animals (*i.e.*, *Trichostrongylus colubriformis* (Giles)) and of plants (*i.e.*, *Heterodera glycines* Ichinohe) [35].

The role of extracellular proteases produced by *B. laterosporus* strain G4 in toxicity to nematodes has been confirmed [60]. The alkaline protease BLG4 was purified from the culture supernatant of the bacterium and assayed against the free-living nematodes *Panagrellus redivivus* Goodey and the plant parasite nematode *Bursaphelenchus xylophilus* (Steiner and Buhrer) Nickle. As observed under scanning electron microscopy, these proteins caused severe damage to the nematode cuticle, with exfoliation of outer layers and the creation of many flaws and scars. The observed effects are reminiscent of those caused by nematophagous fungi whose penetration into nematode body is associated with the action of extracellular proteases that degrade the outer proteinaceus cuticle membrane and the matrix connecting chitin miofibrils to the cuticle of the nematode [61]. This mode of action implying a major role of cuticle degrading proteases, is shared by a community of rhizobacterial species exhibiting nematicidal activity, including *B. laterosporus* [62]. The activity of BLG4 against nematodes was also demonstrated by *in vitro* bioassays employing purified recombinants. The *BLG4* enconding gene was cloned and expressed in *Bacillus subtilis* (Ehrenberg) Cohn and the gene analysis revealed that it showed a high homology with genes encoding for the subtilisin family of serine proteases and for other cuticle-degrading proteases. A significant reduction in pathogenicity was obtained in bioassays employing a *BLG4*-deficient mutant [63]. The involvement of the protease in the bacterial infection process was also demonstrated by its immunofluorescent localization during the degradation of nematode cuticles [64,65]. Although purified proteases were identified as major toxic factors, they were able to cause mortality of nematodes less than the crude extracellular extract, so other virulent factors are implicated [66].

### 3.3. Mollusks

The molluscicidal activity of bacterial strains belonging to the *Brevibacillus brevis* phylogenetic cluster, including *B. laterosporus*, was initially reported against the aquatic snail *Biomphalaria glabrata* (Say), an important vector of flatworm parasites of the genus *Schistosoma*, etiologic agents of the tropical disease schistosomiasis [67]. In the target range include the zebra mussel *Dreissena polymorpha* (Pallas), an invasive bivalve species with severe negative impacts on aquatic ecosystems, with special regard to phytoplankton, zooplankton, vegetation, water chemistry, zoobenthos and fish [68]. The toxicity against mollusks is associated with the whole bacterial culture, and has been specifically associated to the production of bacterial antibiotics such as Gramicidin S and D [69]. A higher susceptibility was observed for animals with a shorter life cycle, and the levels of toxicity against the zebra mussel veliger stage were comparable to that of the entomopathogen *B. thuringiensis* against its targets. 

## 4. Antimicrobial Features

Some *B. laterosporus* strains exhibit a broad-spectrum antimicrobial activity against various bacteria and fungi. This biological property is shared with members of the *Brevibacillus* clade and has been associated with the production of a wide range of enzymes and antibiotics [70].

Growth inhibition of the phytopathogenic fungi *Fusarium oxysporum* f. sp. *ciceri* (Padwick) Matuo & K. Sato, *F.* *semitectum* Berk. and Ravenel, *Magnaporthe grisea* (Hebert) Barr and *Rhizoctonia oryzae* Ryker and Gooch, and of the Gram-positive coccal bacterium *Staphylococcus aureus* Rosenbach was caused by a *B. laterosporus* strain, designated BPM3, isolated from mud in India. The antibacterial and antifungal compounds responsible for the observed effects were purified and characterized. Preliminary information on the chemical composition of the most active fraction was obtained by spectroscopic analysis indicating the presence of C-H, the carbonyl group, the dimethyl group, -CH_2_ and the methyl group [71].

Evidence of fungal inhibition was obtained with the *B. laterosporus* isolate ZQ2 against various apple tree phytopathogens including *Rhizoctonia solani* Kuhn, *Fusarium oxysporum* Schlecht, *F. solani* (Mart.), *Aspergillus fumigatus* Fres, *Alternaria alternata* (Fr.) Keissler, *Valsa sordida* Nits, *Colletotrichum gloeosporioides* (Penz.) Penz. and Sacc., *Botrytis* *cinerea* Pers. and *Physalospora piricola* Nose. This strain was isolated from an apple tree rhizosphere in China. The antifungal activity of the culture filtrate, achieving an inhibition level of around 80%, was maintained after heat treatments up to 120 °C for 30 min, ultraviolet irradiations or pH variations from 1 to 11 [72].

*B. laterosporus* has long been noted for its antifungal properties when isolated from rhizosphere soil samples [73] and features among plant growth promoting rhizobacteria (PGPR) [74]. In specific experiments, *B. laterosporus* preparations promoted lettuce seedling growth [75]. 

These properties have been associated with the production of antimicrobial peptides, and an agar diffusion bioassay for their bioactivity quantification has been developed [76]. More recently, a novel antimicrobial peptide with a molecular mass of about 1,600 Da from *B. laterosporus* strain A60 was isolated and characterized [77]. This short sequence liner peptide, designated BL-A60, was isolated from the culture broth and its inhibitory activity was determined against diverse plant pathogens including the Gram negative bacteria, *Pseudomonas solanacearum* (Smith) Smith and *Xanthomonas campestris* pv. *vesicatoria* (Doidge) Dye, the Gram-positive bacterium, *Bacillus subtilis* (Ehrenberg) Cohn, and the fungi, *Phytophthora capsici* Leonian, *B. cinerea*, *Verticillium dahliae* Kleb, *F. oxysporum*. The antimicrobial activity was thermostable, being maintained at 100 °C for 15 min after treatment, and was not affected by pH variations from 3 to 11, or after treatments with protease k, trypsin and pepsin. Among the chemical properties is the fact that it is a cationic peptide, soluble either in water or in organic solvents (e.g., acetonitrile), which indicates an amphipathic behavior.

It is debated that the action of antimicrobial peptides against their microbial targets implies the interaction with the cell membrane where the formation of ion channels and transmembrane pores leads to cell rupture and lysis. Eventually, peptides might enter the cell and be translocated in the cytoplasm where they can disturb protein synthesis by interacting with DNA and RNA [78]. The BL‑A60 might act in a similar way, but these aspects have not been clarified so far.

Some strains of *B. laterosporus* also produce chitinases that may play a major role in the degradation of the cell wall of fungi. A new *B. laterosporus* isolate from mangrove marsh soil in India, designated Lak1210, when grown on media containing colloidal chitin, produces chitinases that are released in the culture supernatant. The activity of these antifungal proteins was assayed against the phytopathogenic fungus *Fusarium equiseti* (Corda) Sacc. These proteins show high homology with a 89.4-kDa four domain chitodextrinase and with a 69.4-kDa two domain chitinase (ChiA1) encoded by *B. laterosporus* LMG15441 [79]. Another recently discovered broad-spectrum antimicrobial peptide produced by strain GI-9 is laterosporulin, a thermo-stable peptide with a molecular mass of 5.6 kDa [80].

Besides phytopathogenic bacteria and fungi, the antimicrobial properties of *B. laterosporus* were also demonstrated *in vitro* against the bacterium *Paenibacillus larvae* (White), the causal agent of American foulbrood of honeybees [81].

The antifungal and antibacterial properties of certain *B. laterosporus* strains have found interest in medicine for their production of antibiotics with therapeutic effects. An example is represented by the antibiotic laterosporamine that shows *in vitro* and *in vivo* inhibitory effects against a broad spectrum of Gram-positive and Gram-negative bacteria including *B. subtilis*, *Bacillus anthracis* Cohn, *Staphylococcus aureus* Rosenbach, *Streptococcus pneumoniae* (Klein) Chester, *Streptococcus pyogenes* Rosenbach, *Escherichia coli* (Migula) Castellani and Chalmers, *Klebsiella pneumoniae* (Schroeter) Trevisan, *Salmonella typhimurium* (Loeffler) Castellani and Chalmers, *Pseudomonas aeruginosa* (Schroeter) Migula [82].

A *B. laterosporus* strain isolated from tropical sea water at the Papua New Guinea coast was found to produce in culture the acyldipeptides tupuseleiamides and the antifungal polyketides basiliskamides, which significantly inhibited the bacterium *E. coli* and the fungus *Candida albicans* (Robin) Berkhout [83]. The same marine isolate (PBG-276) was reported to produce various antibiotics including loloatins, bogorols and the lipopeptide tauramamide [84]. 

Among other antibiotics released in the culture broth by *B. laterosporus* are the cyclodecapeptide laterocidin [85], the thrombin inhibitors bacithrocins A, B and C [86], the aminopeptidase M inhibitor leuhistin [87] and the antitumor antibiotic spergualin [30]. Other strains produce the industrially important enzyme cephalosporin acylase, useful in the cephalosporin derivatives production process. The strains were isolated from soil [88].

Owing to the antagonism against various noxious microorganisms, *B. laterosporus* is reported to produce beneficial effects also inside mammalian intestines after being administered as a feed additive [28]. As a result of these probiotic effects, the oral administration of *B. laterosporus* spores to poultry can improve feed conversion and weight gain [29,89].

## 5. Other Properties and Uses

Due to the production of various metabolites, *B. laterosporus* bioactivity is not limited to invertebrates, bacteria and fungi. Among other possible applications, algicidal effects against the harmful cyanophytes belonging to different genera including *Oscillatoria*, *Anabaena*, *Microcystis* and *Nostoc*, have been demonstrated [90,91]. 

Another field of growing interest is the use of microorganisms for bioremediation that is the biotreatment of a contaminated site for the degradation and removal of pollutant or contaminant compounds. The list of substances that can be degraded by *B. laterosporus* strains is continuously growing. Representative examples are the biodegradation of polyvinyl alcohol to acetate [92], the decolorization of different textile azo dyes through the production of diverse enzymes including lignin peroxidase, laccase, aminopyrine *N*-demethylase, NADH-DCIP reductase and malachite green reductase [93], the biodegradation of vegetable tannins in tannery effluents [94], the biodegradation of toluene and phenol [95], the biosorption of toxic metals from aqueous solutions [96] and metal detoxification in wastewater systems [97,98].

## 6. Conclusions

Wide-ranging studies have emphasized the potential of *B. laterosporus* as a biological control agent against insects, nematodes, mollusks and plant pathogens. In addition, owing to its antimicrobial features and to the production of specific antibiotic compounds, this species have found use in medicine. More recently, as a result of its biodegradation and decontamination properties, its use in bioremediation has also been proposed (Table 1).

**Table 1:** Summary of the range of activity of *Bravibacillus laterosporus* and some of the active compounds produced by different isolates.

Active compounds	Main activity	References
Complementary toxins ISP1 and ISP2 (=Vip1Da1; Vip2Ad1)	Insecticidal (Coleoptera)	[40,41]
Complementary toxins MIS and RAR (=Vip1Ba1 and Vip2Ba1)	Insecticidal (Coleoptera)	[42]
Insecticidal crystal proteins (PBs)	Mosquitocidal	[49]
Proteins from spores and CSPB	Insecticidal (mosquitoes, flies)	[34,43,45]
Low MW (2,900 Da) protein	Nematicidal	[59]
Alkaline protease BLG4	Nematicidal	[60,61,62,63,64,65]
Chitinases (*i.e.*, ChiA1, chitodextrinase)	Insecticidal, fungicidal	[79]
Gramicidin S and D	Molluscicidal	[69]
Diverse antimicrobial compounds	Antibacterial, antifungal	[71,72]
BL-A60 Antimicrobial peptide	Antibacterial, antifungal	[77]
Laterosporulin	Antibacterial	[80]
Antibiotics and drugs (*i.e.*, laterosporamine, tupuseleiamides, basilikamides, loloatins, bogorols, taraumide, laterocidin, bacithrocins A-B-C, leuhistin, spergualin, cephalosporin acylase)	Antibacterial, antifungal, other therapeutic effects	[30,82,83,84,85,86,87]
Enzymes (*i.e.*, lignin peroxidase, laccase, aminopyrine N-demethylase, NADH-DCIP reductase and malachite green reductase)	Decontamination, detoxification, bioremediation	[88,92,93,94,95,96,97,98]

After the first diversity studies conducted to compare the genomes of different *B. laterosporus* strains [99], more recently the whole genome sequencing of strains LMG 15441 and GI-9 generated valuable information on the genomic determinants of the useful activities of this bacterium [26,27]. The potential to produce a variety of polyketides and nonribosomial peptides was highlighted, corroborating the results of studies on its antimicrobial and antibiotic features. In addition, different putative genes encoding potential virulence factors were detected. Among these, there are genes encoding collagenase, chitinases, immune inhibitors, and insecticidal toxins showing similarities to other bacterial biocides, as produced by *Lysinibacillus sphaericus* Meyer and Neide. 

Interestingly, despite a high genotypic homology among strains, differences in phenotypic properties have been detected, which is in line with a variable activity range expressed by different strains. Decades of studies with the best-established insect pathogen, *B. thuringiensis*, have uncovered a wide diversity of insecticidal toxins being synthesized by diverse strains. Similarly, new *B. laterosporus* isolates are continuously being collected worldwide and efforts are being conducted to purify and characterize novel bacterial enzymes and compounds for biotechnological exploitation [77,79,80,100,101]. However, the mechanism of action of many compounds is still undetermined and further investigation is needed. Future screening studies will also reveal other possible and useful applications of this multifunctional species.

## References

[1] Oliveira E.J., Rabinovitch L., Monnerat R.G., Passos L.K., Zahner V. (2004). Molecular characterization of *Brevibacillus laterosporus* and its potential use in biological control. Appl. Environ. Microbiol..

[2] Khan M.R., Saha M.L., Afroz H. (2001). Microorganisms associated with gemstones. Bangladesh J. Bot..

[3] Raymundo A.K., Capistrano B.G., Aquino A. (1997). Isolation, characterization and identification of bacteria from lahar. Philipp. Agric. Sci..

[4] Laubach A.C. (1916). Studies on aerobic, sporebearing, non pathogenic bacteria. Spore bearing organism in water. J. Bacteriol..

[5] Suslova M.Y., Lipko I.A., Mamaeva E.V., Parfenova V.V. (2012). Diversity of cultivable bacteria isolated from the water column and bottom sediments of the Kara Sea shelf. Mikrobiologiia (Russ. Federation).

[6] White G.F. (1912). The cause of European foulbrood. US Dep. Agric. Bur. Entomol..

[7] Roy D.K., Singh G.P., Sahay A., Sahay D.N., Suryanarayana N. (2006). Leaf surface microflora for tasar crop improvement. Indian Silk.

[8] Sarkar P.K., Hasenack B., Nout M.J.R. (2002). Diversity and functionality of *Bacillus* and related genera isolated from spontaneously fermented soybeans (Indian Kinema) and locust beans (African Soumbala). Int. J. Food Microbiol..

[9] Adegunloye D.V., Adetuyi F.C., Akinyosoye F.A., Doyeni M.O. (2007). Microbial analysis of compost using cowdung as booster. Pak. J. Nutr..

[10] Varadaraj M.C., Devi N., Keshava N., Manjrekar S.P. (1993). Antimicrobial activity of neutralized extracellular culture filtrates of lactic acid bacteria isolated from a cultured Indian milk product ('dahi'). Int. J. Food Microbiol..

[11] Román-Blanco C., Sanz-Gómez J.J., López-Díaz T.-M., Otero A., García-López M.-L. (1999). Numbers and species of *Bacillus* during the manufacture and ripening of Castellano cheese. Milchwissenschaft.

[12] Iurlina M.O., Fritz R. (2005). Characterization of microorganisms in Argentinean honeys from different sources. Int. J. Food Microbiol..

[13] Fangio M.F., Roura S.I., Fritz R. (2010). Isolation and identification of *Bacillus* spp. and related genera from different starchy foods. J. Food Sci..

[14] Sharma A., Rao C.L.S.N., Ball B.K., Hasija S.K. (1996). Characteristics of extracellular proteases produced by *Bacillus laterosporus* and *Flavobacterium* sp. isolated from gelatin-factory effluents. World J. Microbiol. Biotechnol..

[15] Chen Y., Gao H., Zhang Y., Deng M., Wu Z., Zhu L., Duan Q., Xu B., Liang C., Yue Z., Xiao X. (2012). Analysis of the bacterial diversity existing on animal hide and wool: Development of a preliminary PCR-restriction fragment length polymorphism fingerprint database for identifying isolates. J. AOAC Int..

[16] Bagherzadeh Kasmani F., Karimi Torshizi M.A., Allameh A., Shariatmadari F. (2012). A novel aflatoxin-binding *Bacillus* probiotic: Performance, serum biochemistry, and immunological parameters in Japanese quail. Poultry Sci..

[17] McCray A.H. (1917). Spore-forming bacteria of the apiary. J. Agric. Res..

[18] Smith N.R., Gordon R.E., Clark F.E. (1952). Aerobic sporeforming bacteria. US Dep. Agric. Agric. Monograph..

[19] Steinhaus E.A. (1946). An orientation with respect to members of the genus *Bacillus* pathogenic for insects. Bacteriol. Rev..

[20] Shida O., Takagi H., Kadowaki K., Komagata K. (1996). Proposal for two new genera, *Brevibacillus* gen. nov. and *Aneurinobacillus* gen. nov. Int. J. Syst. Bacteriol..

[21] Ruiu L., Satta A., Floris I. (2013). Emerging entomopathogenic bacteria for insect pest management. Bull. Insectology.

[22] Gilliam M., Valentine D.K. (1976). Bacteria isolated from the intestinal contents of foraging worker honey bees, *Apis mellifera*: The Genus *Bacillus*. J. Invertebr. Pathol..

[23] Bailey L. (1963). The pathogenicity for honey-bee larvae of microorganisms associated with European foulbrood. J. Insect Pathol..

[24] Alippi A.M. (1991). A comparison of laboratory techniques for the detection of significant bacteria of the honey bee, *Apis mellifera*, in Argentina. J. Apicult. Res..

[25] Shimanuki H., Knox D.A. (1991). Diagnosis of honey bee diseases. US Dep. Agric. Agric. Handbook.

[26] Djukic M., Poehlein A., Thürmer A., Daniel R. (2011). Genome sequence of *Brevibacillus laterosporus* LMG 15441, a pathogen of invertebrates. J. Bacteriol..

[27] Sharma V., Singh P.K., Midha S., Ranjan M., Korpole S., Patil P.B. (2012). Genome sequence of *Brevibacillus laterosporus* strain GI-9. J. Bacteriol..

[28] Hong H.A., Duc L.H., Cutting S.M. (2005). The use of bacterial spore formers as probiotics. FEMS Microbiol. Rev..

[29] Porubcan R.S. (2003). Administering *Bacillus laterosporus* to increase poultry feed conversion and weight gain. U.S. Patent.

[30] Umezawa K., Takeuchi T. (1987). Spergualin: A new antitumour antibiotic. Biomed. Pharmacother..

[31] Hannay C.L. (1957). The parasporal body of *Bacillus laterosporus* Laubach. J. Biophys. Biochem. Cytol..

[32] Fitz-James P.C., Young I.E. (1958). Morphological and chemical studies of the spores and parasporal bodies of *Bacillus laterosporus*. J. Biophys. Biochem. Cytol..

[33] Montaldi F.A., Roth I.L. (1990). Parasporal bodies of *Bacillus laterosporus* sporangia. J. Bacteriol..

[34] Rivers D.B., Vann C.N., Zimmack H.L., Dean D.H. (1991). Mosquitocidal activity of *Bacillus laterosporus*. J. Invertebr. Pathol..

[35] Singer S. (1996). The Utility of Morphological Group II *Bacillus*. Adv. Appl. Microbiol..

[36] Salama H.S., Foda M.S., El-Bendary M.A., Abdel-Razek A. (2004). Infection of red palm weevil, *Rhynchophorus ferrugineus*, by spore-forming bacilli indigenous to its natural habitat in Egypt. J. Pest Sci..

[37] Echeverri-Molina D., Santolamazza-Carbone S. (2010). Toxicity of synthetic and biological insecticides against adults of the Eucalyptus snout-beetle *Gonipterus scutellatus* Gyllenhal (Coleoptera: Curculionidae). J. Pest Sci..

[38] Du Rand N., Laing M.D. (2011). Determination of insecticidal toxicity of three species of entomopathogenic spore-forming bacterial isolates against *Tenebrio molitor* L. (Coleoptera: Tenebrionidae). Afr. J. Microbiol. Res..

[39] Arnaut G., Boets A., Damme N., Van Rie J. (2011). Toxins. U.S. Patent.

[40] Warren G.W., Carozzi N.B., Koziel M.G. (1997). Vegetative insecticidal proteins: Novel proteins for control of corn pests. Advances in Insect Control: The Role of Transgenic Plants.

[41] *Bacillus thuringiensis* Toxin Nomenclature. http://www.lifesci.sussex.ac.uk/home/Neil_Crickmore/Bt/.

[42] Schnepf H.E., Narva K.E., Stockhoff B.A., Lee S.F., Walz M., Sturgis B. (2005). Pesticidal toxins and genes from *Bacillus laterosporus* strains. U.S. Patent.

[43] Favret E.M., Yousten A.A. (1985). Insecticidal activity of *Bacillus laterosporus*. J. Invertebr. Pathol..

[44] Erturk O., Demirbag Z. (2006). Studies on bacterial flora and biological control agent of *Cydia pomonella* L. (Lepidoptera: Tortricidae). Afr. J. Biotechnol..

[45] Ruiu L., Floris I., Satta A., Ellar D.J. (2007). Toxicity of a *Brevibacillus laterosporus* strain lacking parasporal crystals against *Musca domestica* and *Aedes aegypti*. Biol. Contr..

[46] Smirnova T.A., Minenkova I.B., Orlova M.V., Lecadet M.-M., Azizbekyan R.R. (1996). The crystal-forming strains of *Bacillus laterosporus*. Res. Microbiol..

[47] Orlova M.V., Smirnova T.A., Ganushkina L.A., Yacubovich V.Y., Azizbekyan R.R. (1998). Insecticidal activity of *Bacillus laterosporus*. Appl. Environ. Microbiol..

[48] Goldberg L.J., Margalit J. (1977). A bacterial spore demonstrating rapid larvicidal activity against *Anopheles sergentii*, *Uranotaenia unguiculata*, *Culex univittatus*, *Aedes aegypti* and *Culex pipiens*. Mosq. News.

[49] Zubasheva M.V., Ganushkina L.A., Smirnova T.A., Azizbekyan R.R. (2010). Larvicidal activity of crystal-forming strains of *Brevibacillus laterosporus*. Appl. Biochem. Microbiol..

[50] Zubasheva M.V., Ganushkina L.A., Smirnova T.A., Azizbekyan R.R. (2011). Enhancement of larvicidal activity of *Brevibacillus laterosporus* by bioincapsulation in Protozoa *Tetrahymena pyriformis* and *Entamoeba moshkovskii*. Appl. Biochem. Microbiol..

[51] Ruiu L., Delrio G., Ellar D.J., Floris I., Paglietti B., Rubino S., Satta A. (2006). Lethal and sub-lethal effects of *Brevibacillus laterosporus* on the housefly (*Musca domestica*). Entomol. Exp. Appl..

[52] Bravo A., Gill S.S., Soberon M. (2007). Mode of action of *Bacillus thuringiensis* Cry and Cyt toxins and their potential for insect control. Toxicon.

[53] Ruiu L., Satta A., Floris I. (2012). Observations on house fly larvae midgut ultrastructure after *Brevibacillus laterosporus* ingestion. J. Invertebr. Pathol..

[54] Ruiu L., Satta A., Floris I. Ultrastructural changes in the gut of adult flies after *Brevibacillus laterosporus* ingestion. Proceedings of the 43rd Annual Meeting of the Society for Invertebrate Pathology.

[55] Ruiu L., Satta A., Floris I. (2008). Immature house fly (*Musca domestica*) control in breeding sites with a new *Brevibacillus laterosporus* formulation. Environ. Entomol..

[56] Ruiu L., Satta A., Floris I. (2011). Comparative applications of azadirachtin and *Brevibacillus laterosporus* based formulations for house fly management experiments in dairy farms. J. Med. Entomol..

[57] Ruiu L., Satta A., Floris I. (2007). Susceptibility of the house fly pupal parasitoid *Muscidifurax raptor* (Hymenoptera: Pteromalidae) to the entomopathogenic bacteria *Bacillus thuringiensis* and *Brevibacillus laterosporus*. Biol. Contr..

[58] Ruiu L., Floris I., Satta A. (2007). Susceptibility of the honeybee (*Apis mellifera* L.) to entomopathogenic bacterial toxins used for the biological control. Redia.

[59] Bone L.W., Singer S. (1991). Control of parasitic nematode ova/larvae with a *Bacillus laterosporus*. U.S. Patent.

[60] Huang X., Tian B., Niu Q., Yang J., Zhang L., Zhang K. (2005). An extracellular protease from *Brevibacillus laterosporus* G4 without parasporal crystals can serve as a pathogenic factor in infection of nematodes. Res. Microbiol..

[61] Maizels R.M., Blaxter M.L., Selkir M.E. (1993). Forms and functions of nematode surfaces. Exp. Parasitol..

[62] Lian L.H., Tian B.Y., Xiong R., Zhu M.Z., Xu J., Zhang K.Q. (2007). Proteases from *Bacillus*: A new insight into the mechanism of action for rhizobacterial suppression of nematode populations. Lett. Appl. Microbiol..

[63] Tian B., Li N., Lian L., Liu J., Yang J., Zhang K.Q. (2006). Cloning, expression and deletion of the cuticle-degrading protease BLG4 from nematophagous bacterium *Brevibacillus laterosporus* G4. Arch. Microbiol..

[64] Tian B., Ke C.R., Huang W., Zhang K.-Q., Huang J.-Z. (2009). Direct visualization of bacterial infection process in nematode hosts by an improved immunocytochemical method. World J. Microbiol. Biotechnol..

[65] Tian B., Huang W., Huang J., Jiang X., Qin L. (2009). Investigation of protease-mediated cuticle-degradation of nematodes by using an improved immunofluorescence-localization method. J. Invertebr. Pathol..

[66] Tian B., Yang J., Lian L., Wang C., Li N., Zhang K.Q. (2007). Role of an extracellular neutral protease in infection against nematodes by *Brevibacillus laterosporus* strain G4. Appl. Microbiol. Biotechnol..

[67] Singer S., Bair T.B., Hammill T.B., Berte A.M., Correa-Ochoa M.M., Stambaugh A.D. (1994). Fermentation and toxin studies of the molluscicidal strains of *Bacillus brevis*. J. Ind. Microbiol..

[68] Strayer D.L., Hattala K.A., Kahnle A.W. (2004). Effects of an invasive bivalve (*Dreissena polymorpha*) on fish in the Hudson River estuary. Can. J. Fish. Aquat. Sci..

[69] Singer S., Van Fleet A.L., Viel J.J., Genevese E.E. (1997). Biological control of the zebra mussel *Dreissena polymorpha* and the snail *Biomphalaria glabrata*, using gramicidin S and D and molluscicidal strains of *Bacillus*. J. Ind. Microbiol. Biotechnol..

[70] Chandel S., Allan E.J., Woodward S. (2010). Biological control of *Fusarium oxysporum* f.sp. *lycopersici* on tomato by *Brevibacillus brevis*. J. Phytopathol..

[71] Saikia R., Gogoi D.K., Mazumder S., Yadav A., Sarma R.K., Bora T.C., Gogoi B.K. (2011). *Brevibacillus laterosporus* strain BPM3, a potential biocontrol agent isolated from a natural hot water spring of Assam, India. Microbiol. Res..

[72] Song Z., Liu K., Lu C., Yu J., Ju R., Liu X. (2011). Isolation and characterization of a potential biocontrol *Brevibacillus laterosporus*. Afr. J. Microbiol. Res..

[73] Idris H.A., Labuschagne N., Korsten L. (2008). Suppression of *Pythium ultimum* root rot of sorghum by rhizobacterial isolates from Ethiopia and South Africa. Biol. Contr..

[74] Zhang S., Reddy M.S., Kokalis-Burelle N., Wells L.W., Nightengale S.P., Kloepper J.W. (2001). Lack of induced systemic resistance in peanut to late leaf spot disease by plant growth-promoting rhizobacteria and chemical elicitors. Plant Dis..

[75] Yobo K.S., Laing M.D., Hunter C.H. (2004). Effect of commercially available rhizobacteria strains on growth and production of lettuce, tomato and pepper. S. Afr. J. Plant Soil.

[76] Li X., Wang Z., Dong X., Wang G., Jia Y. Bioactivity quantification of a novel antimicrobial peptide by agar diffusion bioassay. Proceedings of the 2011 International Conference on New Technology of Agricultural Engineering.

[77] Zhao J., Guo L., Zeng H., Yang X., Yuan J., Shi H., Xiong Y., Chen M., Han L., Qiu D. (2012). Purification and characterization of a novel antimicrobial peptide from *Brevibacillus laterosporus* strain A60. Peptides.

[78] Brogden K.A. (2005). Antimicrobial peptides: Pore formers or metabolic inhibitors in bacteria?. Nat. Rev. Microbiol..

[79] Prasanna L., Eijsink V.G.H., Meadow R., Gaseidnes S. (2013). A novel strain of *Brevibacillus laterosporus* produces chitinases that contribute to its biocontrol potential. Appl. Microbiol. Biotechnol..

[80] Singh P.K., Chittpurna, Ashish, Sharma V., Patil P.B., Korpole S. (2012). Identification, purification and characterization of laterosporulin, a novel bacteriocin produced by *Brevibacillus* sp. strain GI-9. PLoS One.

[81] Alippi A.M., Reynaldi F.J. (2006). Inhibition of the growth of *Paenibacillus larvae*, the causal agent of American foulbrood of honeybees, by selected strains of aerobic spore-forming bacteria isolated from apiarian sources. J. Invertebr. Pathol..

[82] Shoji J., Sakazaki R., Wakisaka Y., Koizumi K., Mayama M. (1976). Isolation of a new antibiotic, laterosporamine. (Studies on antibiotics from the genus *Bacillus*. XIII). J. Antibiot..

[83] Barsby T., Kelly M.T., Andersen R.J. (2002). Tupuseleiamides and basiliskamides, new acyldipeptides and antifungal polyketides produced in culture by a *Bacillus laterosporus* isolate obtained from a tropical marine habitat. J. Nat. Prod..

[84] Desjardine K., Pereira A., Wright H., Matainaho T., Kelly M., Andersen R.J. (2007). Tauramamide, a lipopeptide antibiotic produced in culture by *Brevibacillus laterosporus* isolated from a marine habitat: Structure elucidation and synthesis. J. Nat. Prod..

[85] Qin C., Xu C., Zhang R., Niu W., Shang X. (2010). On-resin cyclization and antimicrobial activity of Laterocidin and its analogues. Tetrahedron Lett..

[86] Kamiyama T., Umino T., Nakamura Y., Itezono Y., Sawairi S., Satoh T., Yokose K. (1994). Bacithrocins A, B and C, novel thrombin inhibitors. J. Antibiot..

[87] Aoyagi T., Yoshida S., Matsuda N., Ikeda T., Hamada M., Takeuchi T. (1991). Leuhistin, a new inhibitor of aminopeptidase M, produced by *Bacillus laterosporus* BMI156–14F1. I. Taxonomy, production, isolation, physico-chemical properties and biological activities. J. Antibiot..

[88] Aramori I., Fukagawa M., Tsumura M., Iwami M., Yokota Y., Kojo H., Kohsaka M., Ueda Y., Imanaka H. (1991). Isolation of soil strains producing new cephalosporin acylases. J. Ferment. Bioeng..

[89] Wolfenden R.E., Pumford N.R., Morgan M.J., Shivaramaiah S., Wolfenden A.D., Pixley C.M., Green J., Tellez G., Hargis B.M. (2011). Evaluation of selected direct-fed microbial candidates on live performance and *Salmonella* reduction in commercial turkey brooding houses. Poultry Sci..

[90] Kuznetsova N.I., Azizbekyan R.R., Konyukhov I.V., Pogosyan S.I., Rubin A.B. (2008). Inhibition of photosynthesis in cyanobacteria and plankton algae by the bacterium *Brevibacillus laterosporus* metabolites. Dokl. Biochem. Biophys..

[91] Wen J., XiangHu H., ChangLing L., JiaHui Z. (2013). Research on algicidal effect of bioactive metabolites of *Brevibacillus laterosporus* on *Oscillattoria* sp. in shrimp pond. J. Fish. Chin..

[92] Lim J.G., Park D.H. (2001). Degradation of polyvinyl alcohol by *Brevibacillus laterosporus*: Metabolic pathway of polyvinyl alcohol to acetate. J. Microbiol. Biotechnol..

[93] Gomare S.S., Govindwar S.P. (2009). *Brevibacillus laterosporus* MTCC 2298: A potential azo dye degrader. J. Appl. Microbiol..

[94] Jeyaseelan A., Sivashanmugam K., Jayaraman K. (2008). Comparative applications of bioreactor and shake flask system for the biodegradation of tannin and biotreatment of composite tannery effluents. Pollut. Res..

[95] Reda A.B., Ashraf T.A.-H. (2010). Optimization of bacterial biodegradation of toluene and phenol under different nutritional and environmental conditions. J. Appl. Sci. Res..

[96] Zouboulis A.I., Loukidou M.X., Matis K.A. (2004). Biosorption of toxic metals from aqueous solutions by bacteria strains isolated from metal-polluted soils. Process Biochem..

[97] Holail H., Al-Bahadly A., Olama Z. (2011). Detoxification of hexavalent chromium Cr(VI) by *Bacillus laterosporus* and its application in Lebanese waste water. WIT Trans. Ecol. Environ..

[98] Kamika I., Momba M.N.B. (2011). Comparing the tolerance limits of selected bacterial and protozoan species to nickel in wastewater systems. Sci. Total Environ..

[99] Zahner V., Rabinovitch L., Suffys P., Momen H. (1999). Genotypic diversity among *Brevibacillus laterosporus* strains. Appl. Environ. Microbiol..

[100] Arulmani M., Aparanjini K., Vasanthi K., Arumugam P., Arivuchelvi M., Kalaichelvan P.T. (2007). Purification and partial characterization of serine protease from thermostable alkalophilic *Bacillus laterosporus*-AK1. World J. Microbiol. Biotechnol..

[101] Usharani B., Muthuraj M. (2010). Production and characterization of protease enzyme from *Bacillus laterosporus*. Afr. J. Microbiol. Res..

